# Brokerage System for Integration of LrWPAN Technologies

**DOI:** 10.3390/s22051733

**Published:** 2022-02-23

**Authors:** Josiah E. Balota, Ah-Lian Kor

**Affiliations:** School of Built Environment, Engineering, and Computing, Leeds Beckett University, Leeds LS6 3QS, UK; josiahbalota@gmail.com

**Keywords:** Bluetooth Low Energy, Zigbee, Thread, integration, interoperability, WSN, IEEE 802.15.4, Internet of Things (IoT), wireless sensor networks, wireless broker, LrWPAN, low-power, low-rate

## Abstract

The prevalent demand for remote data sharing and connectivity has catalysed the development of many wireless network technologies. However, low-power and low-rate wireless network technologies have emerged as the preferred choice (due to cheap procurement and maintenance cost, efficiency, and adaptability). Currently, these groups of wireless networks are adopted in homes, health, and business sectors. The increase in existing WSNs has resulted in the incompatibility of wireless network protocols and poses a problem that results in high acquisition or maintenance costs, increased complexity, reliability inadequacies in some instances, lack of uniformity within similar standards, and high energy consumption. To address this problem, we develop a novel machine-to-machine software-based brokerage application (known as JosNet) for interoperability and integration between Bluetooth LE, Zigbee, and Thread wireless network technologies. JosNet allows one network protocol to exchange data packets or commands with each other. In this paper, we present a novel working network brokerage model for a one-to-one network protocol to communication (e.g., from Zigbee to Bluetooth) or one-to-many network protocol communication (e.g., from Bluetooth to Zigbee, Thread, etc.) to securely send messages in a large-scale routing process for short or long-range connections. We also present a large-scale implementation of JosNet using a routing table for large areas. The results show an industry standard performance for end-to-end latency time and throughput.

## 1. Introduction

Within the last decade, the prolific deployment of smart technologies has put demands on enhanced performance accompanied by reduced cost or resources [1,2]. However, diverse, and noninteroperable smart technologies pose great challenges to the general smart technology community. Devices operating on similar standards such as the IEEE 802.15.4, has enormous diverse implementation platforms and consequently, developers face the problem of deciding which system protocol is best suited to their requirements. Numerous low-rate wireless networks for smart devices are subject to similar installation procedures. Wireless Sensor Networks (WSN) is expanding rapidly with new protocols and network versions due to an increase in the volume of network traffic [1,3]. Even though performance is increasingly efficient with greatly reduced power consumption, disparity amongst network protocols is growing exponentially [4]; this consequently raises several network compatibility-related issues. Incompatible and diverse architecture specifications [5] pose a dysfunctional communication barrier that incurs high costs to end-users, creates security risks, and increases installation complexity for field workers, as well as distrust for the systems [6]. However, these could only be addressed through heterogeneous networks integration. To date, there is limited existing interoperability for low-rate wireless sensor networks [1]. Reference [7] discussed the barriers to the interoperability of M2M communication between low-rate wireless networks and IoT devices. However, they failed to provide concrete solutions to address the disparity in low-rate wireless communication. Reference [8] maintained that WSN is increasingly gaining prominence, thereby increasing the complexity of interoperability around WSN and internet protocol (IP). To reiterate, there are similar instances where problems are identified without any physical solution to solving and testing the interoperability of all networks [9,10]. Undeniably, generality, as well as efficiency, should be two primary goals when unifying wireless sensor networks.

In the current market, smart system-on-chips (SoC) such as the EFR32xG21 wireless Gecko starter kit and NXP USB-KW41Z dongle aim to integrate multiple wireless networks. These SoC devices are designed for developers so that they can install or configure more than one WSN on small chips which cannot communicate with each other. However, our research will address this challenge. To summarise, several identified gaps that are addressed in this research are the adoption of an integrated heterogeneous protocol for Wireless Sensor Networks (WSN) that are compatible with similar standards where diverse WSN protocols would perform as a single entity that “understands” each other. Such integrated protocol can be adopted by various network technologies defined under the IEEE 802.15.4 low-rate standard in a less complex, simplified form. An integration brokerage is a third-party mediator that facilitates communication between two systems [11,12,13]. JosNet (our novel research deliverable) is a brokerage system that facilitates the integration and interoperability of low-rate and low-power WSN protocols. The underlying mechanism includes bridging the gap at the OSI layers of the different wireless network communication protocols, using OSI layer protocol switching as a brain-box to relay data or packets from one wireless technology to the other, therefore “understanding” each other. In the next section, we shall summarise the aim and research objectives. BLE is not classed under IEEE 802.15.4 but, rather, under the IEEE 802.15.1 standard; it is, however, necessary to include Bluetooth LE, as it is a major player in the IoT industry.

### 1.1. Aim and Research Objectives

To reiterate, the aim of this research is to design and implement a heterogeneous protocol using physical real hardware that integrates low-rate and low-power WSN protocols beginning at the physical layer of the OSI model into an interoperable platform for IoT devices to enhance network adaptability, reliability, energy efficiency, and cost-effectiveness. The following list of research objectives support this aim:

- RO1: Integrate several low-energy low-rate wireless network protocols defined by IEEE 802.15.4 (e.g., Zigbee and Thread) and Bluetooth Low Energy through a novel broker (known as JosNet).
- RO2: Develop a network data framework using a sequence diagram for data transmission architecture to highlight the handing-over procedure from one network to the other (i.e., Bluetooth Low Energy to Zigbee) by JosNet as an integration interpreter.
- RO3: Performance Monitoring
  ∘ RO3.1: Develop a time synchronising mechanism using an end-to-end device IP address and port number to track or monitor network delay between the source and destination nodes;
  ∘ RO3.2: Build a predictive model for the delivery time and data packet size based on raw data collected by JosNet in CSV format;
  ∘ RO3.3: Build a predictive model for throughput based on raw data collected by JosNet in CSV format.
- RO4: Validation
  ∘ RO4.1: Design and implement experiments using JosNet to provide insight into the behaviour of low-energy WSN protocols or standards such as Zigbee, Bluetooth Low Energy, and Thread to validate their interoperability;
  ∘ RO4.2: Implement a large-scale campus-wide interconnection of multiple nodes, involving all network protocols tested during this research study (BLE, Zigbee, and Thread). Making use of a similar principle as the mesh routing feature in IEEE 802.15.4 to advocate the possibility of M2M long-distance mesh connectivity of an interoperable network.

### 1.2. Novel Contribution of Research

In this research, we developed a novel software-based network brokerage (known as JosNet) that provides seamless interoperable communication between various low-rate wireless personal area network (LrWPAN) protocols (i.e., Bluetooth Low Energy, Zigbee, Thread, and WirelessHART) that can read, understand, and interpret packets. JosNet provides single-access commands, transmission requirements, authentication, device ID and addresses, mesh route map, and other communication principles in one software location. A vital outcome of JosNet as a heterogeneous protocol for WSN is the provision of a machine to machine (M2M) routing over a long-distance mesh network without the need for local or internet servers. The brokerage is robust, because it provides the facility to add new protocols without disrupting the existing workflow structure. Additionally, a new exchange structure and workflow pattern at the network layer in the point of exchanging packets have been designed to allow authentication and reliability. In summary, JosNet brokerage has the following set of capabilities: (i) imports various external libraries and repositories (example, those from MS Visual Studio, HEX file from nRF Connect, etc.) to provide the relevant features needed for reliable communication and handing over packets from one node to another node; (ii) decodes and transcribes messages (in the form of packets) to “understand” them; (iii) provides mesh routing procedure from the source device to the destination device; (iv) monitors message authenticity, flow sequence, and report inconsistencies as error messages; (v) tracks and analyse network data size and delivery time for building an authentic prediction model; and (vi) manages and controls the unique features of each network technology and provides necessary adjustments to accommodate them.

This paper is organised as follows: Section 2: Literature Review—discusses the OSI layers and IEEE standard that have the potential to bind almost all LrWPAN together under the IEEE 802.4.15 standard; Section 3: Methodology—discusses the development of JosNet and procedures for the physical implementation of the research using software and hardware; Section 4: Results and Evaluation—results of the integration of Zigbee, Bluetooth, and Thread and discusses end-to-end latency, univariate linear regression and average throughput; and Section 5: Conclusion and Future Work.

## 2. Literature Review

IoT devices are being produced by diverse manufacturers, thereby resulting in heterogeneity, disparity, and network interoperability issues [14,15], which is the main concern for the Wireless Sensor Network (WSN) industry [16,17,18]. Undeniably, the interoperability and integration of wireless networks would be the future for seamless connectivity, which could enhance the reliability and efficiency in information sharing. In this section, we shall review relevant low-rate network standards and protocols, followed by interoperability concepts and hardware.

### 2.1. Low-Rate Network Standards and Protocols

The IEEE 802.15.4 standard forms the basis of interoperability by focusing on the physical (PHY) and data link (or Media Access Control) layers of the OSI model for low-power short-distance wireless networks. The IEEE 802.15.4 standard aims to improve the productivity, reliability, convenience, security, and cost. It is designed for low-power wireless devices and encompasses three categories of frequency: 868 MHz (Europe; data rate—20 kb/s; up to 2 MHz channel width; receive sensitivity of up to −92 dBm; modulation scheme BPSK, ASK, or O-QPSK); 915 MHz (North America; data rate—40 kb/s; up to 2 MHz channel width; receive sensitivity of up to −92 dBm; modulation scheme BPSK, ASK, or O-QPSK); and 2450 MHz (or 2.4 GHz, worldwide; data rate—250 kb/s; up to 5 MHz channel width; receive sensitivity of up to −82 dBm; modulation scheme O-QPSK) [19,20,21,22,23]. IEEE 802.15.4 is subdivided into IEEE 802.15.4a that represents Low-rate WPAN alternative PHY and enhancements and clarifications, respectively [24,25]. However, not all Low-rate Wireless Personal Area Network (LrWPAN) technologies adopt the IEEE 802.15.4 standard in its entirety, due to differing focuses of each manufacturer or vendor, for example, when a manufacturer focuses on long-range and saving power (Sigfox), home automation (ZigBee), or internet access through improvised IPv6 (6LoWPAN). Technologies such as ANT, Z-Wave, or Thread have made some modifications to meet companies’ primary focus or to meet specific user demands [26]. Table 1 shows the mapping of the OSI reference model (for Zigbee, Thread, and WirelessHART) and Bluetooth Low Energy Protocol Stack accompanied with details for each layer. Table 2 shows the variations in the network parameters for Zigbee, Thread, WirelessHART, and Bluetooth Low Energy.

### 2.2. WSN Interoperability

For this research, we adopt the IEEE definition for interoperability: “The ability for two or more systems or components to exchange information and use the information that has been exchanged” [27]. IEEE 802.15.4 has been standardised to bring LrWPAN under a single platform, but this does not address the interoperability challenge. The 6LoWPAN protocol is introduced, which makes use of a compressed version of Internet Protocol version 6 (IPv6) to connect millions of small low-energy WSN devices, because it is lightweight, with a faster processing time and energy efficiency [28]. However, it does not address the interoperability challenge inclusively, which is the goal of this research.

#### 2.2.1. Interoperability of Network Protocols

Qiao and Ma [29] proposed a Zigbee enhancement where a Bluetooth module can be added to a Zigbee network using a simplified physical model. The Bluetooth module or device is connected to the Zigbee network (end device, router, and coordinator) using a Universal Asynchronous Receiver/Transmitter (UART) portable USB terminal without changing the original Zigbee network structure and the ability to control or monitor the network over a handheld smartphone. Although the proposal does not include an implementation, it provides an acceptable conceptual groundwork for the integration of Zigbee and Bluetooth. Additionally, it will induce cost savings via the use of any smartphone for control and monitoring purposes. A connection is fostered via the internet, and this poses a major design drawback and flaws, because it will incur additional data transfer costs, thereby countering the initial idea of cost savings. Another approach to effect cost savings is to set up Bluetooth and Zigbee to communicate over the internet using a single gateway (instead of a gateway for each protocol) [30]. They demonstrated protocols integration by placing two Zigbee radio modules at the gateway where one radio module was to collect data at specific intervals while the other radio module was for streaming data. They argued that this design increased the Zigbee data transmission rate with reduced latency. Currently, smart IoT devices can access the internet using IPv6 or 6LoWPAN [31,32]. However, our research focused on M2M communications over Bluetooth LE, Zigbee, Thread, and IEEE 802.15.4 networks. Additionally, this research aims to build a gateway or switch that allows multiple wireless protocols to understand each other through a brokerage interpreter that “interprets” data or requests sent from one protocol so that it could be understood by the other protocols.

For Phase 1 of our research investigation, we selected Bluetooth and Zigbee, because they are the two most popular network technologies within the WPAN spectrum [33,34]. Additionally, both networks can coexist without any significant compromise in the performance. However, their investigation of the impact of coexistence for WiFi, Bluetooth, and Zigbee on performance reveals a major drop in the data transfer speed of either Wi-Fi, Bluetooth, or ZigBee. These networks operate on different channels but under the same wireless network spectrum. Reference [33] conducted four separate tests that focused on the result of interference for Wi-Fi and ZigBee; Bluetooth and ZigBee; Wi-Fi and Bluetooth; and Wi-Fi, Bluetooth, and ZigBee. Although their research findings show a drop in data transmission speed and drop in connection throughput, other, more recent research argues that interference in Zigbee, Bluetooth, and Wi-Fi has an almost insignificant impact due to the following reasons: firstly, new communication software can prevent interference by switching channels, and secondly, Zigbee and Bluetooth transmit very small data sizes [35,36,37].

The IoT family covers an extensive range of low-energy and low-power wireless sensor networks (WSNs), protocols, and standards that could not be added to JosNet due to research constraints. Some examples are Z-wave, Sigfox, ANT+, 6LoWPAN, INSTEON, Wi-Fi HaLow, LoRaWAN, etc. Among all these wireless protocols, Bluetooth LE (BLE) has been proven to maintain better reliability in power management, throughput, and latency [38]. Next to BLE is 6LoWPAN, which has an adaptive capacity to work with other protocols linked via the internet using specialised IPv6 [39], thereby creating smart protocols that change their interactional behaviour. ZigBee is considered unique, because it is championed in the evolution of the IEEE 802.15.4 standard, which defines the PHY and MAC layers of the OSI model. ZigBee has facilitated the inclusion of additional layers (e.g., Network and Application Layers [40]) to improve its network, as well as enhance the intelligence of the application. ZigBee comes up top among other WSNs technologies in terms of encryption, integrity, and authentication [41], which are some vital features for wireless connectivity. Other related works on WSN interoperability include a proposed gateway by Reference [42] for Zigbee and BLE. Reference [43] proposed the use of smartphones as a universal gateway interface between the internet and smart IoT devices. IoT devices such as Bluetooth, Zigbee, and Wi-Fi are required to send packets over the internet, while the smartphone retrieves the predetermined data before forwarding it to the target destination. For the purpose of our research, we have selected BLE and ZigBee due to their pivotal roles in IoT and potentially unify or integrate the IoT industry.

#### 2.2.2. Related Interoperability Approaches

Here, we shall discuss several interoperability approaches. Multiprotocol Label Switching (MPLS) is a form of data-carrying technique for high-performance telecommunications networks where data are carried from one network node to the next based on short part labels to avoid complex look-up in the routing table [44,45]. MPLS is particularly apt for interoperable solutions, as it transports different types of traffic from within the network and operates between the data link layer and network layer of the OSI model. Thus, it is appropriate for network layer integration of network protocols. The benefits of MPLS for interoperability and the use of multiprotocol switching techniques have been discussed in several pieces of research [46,47,48]. To date, MPLS is being superseded or replaced with better and more secured hardware, such as intelligent routers [49].

Virtual Private LAN Service (VPLS) fosters ethernet-based multipoint-to-multipoint communication over IP or MPLS networks. VPLS is a virtual private network (VPN) technology that allows any-to-any (multipoint) connectivity, unlike other protocols that only allows point-to-point tunnels. It has been designed for secure communication and data sharing, although it is argued that VPLS has more functionalities compared to MPLS [50]. For example, “Exponential-e Applied Innovation” is a company that provides VPLS service to businesses that run very large network traffic [51] accommodating busy traffic. It is noteworthy that the primary focus of VPLS is data security, while MPLS is data interoperability [46]. However, it is possible for redesigning VPLS to improve the interoperability amongst the various network protocols [52,53]. Other switching techniques that could support WSN interoperability include the following: Automatically Switched Optical Network (ASON) [54], Automatic Switch-Transport Network (ASTN) [55], and Generalised Multi-Protocol Label Switching (GMPLS) [56].

#### 2.2.3. Hardware Devices to Support Interoperability

In recent years, several IoT research labs such as Silicon Labs, Redpine Signals, NXP Semiconductors, and ST Microelectronics, have been involved in the implementation of multiprotocol wireless connectivity involving two or more of the following standards/protocols: Bluetooth LE, Bluetooth 5, and IEEE 802.15.4 (i.e., Zigbee and Thread). Existing hardware devices for interoperability are discussed below:

- EFR32xG21 Wireless Gecko Starter Kit: Built with multiprotocol software and wireless SoC with Arm Cortex MCU. Wireless Gecko module has a single multiprotocol chip that “enables developers to create a mesh network and evaluate wireless connections” [57]. First, connect a network to the module (e.g., using Bluetooth). Next, press the “PB1” button to switch between protocols—a physical button must be pressed; wait for 10–15 s to give the SoC time to readjust to the next network protocol. A unique feature of the EFR32xG21 series is the ability to manage the coexistence between Wi-Fi, Zigbee, Thread, and Bluetooth networks using the Packet Traffic Arbitration technique to avoid interference with adjacent radios between protocols. However, each network protocol operates independently and does not share data or communicate together.
- NXP USB-KW41Z dongle: A USB development board used as a packet sniffer to monitor over-the-air communication. Developers could configure packet sniffing and build customised gateways on targeted devices using Bluetooth or IEEE 802.15.4 protocols. The transmission frequency is 2.4 GHz, with ultra-low power. It is a multi-protocol MCU and not designed as a gateway interpreter or interoperable protocol. A development board facilitates monitoring of network communication in Bluetooth smart, BLE, and/or IEEE 802.15.4 protocols, since only one network protocol can operate at one single time [58,59].
- Redpine Signals RS9116/RS9113: A multiprotocol wireless SoC and modules n-Link, managed and controlled by an internal protocol arbitration manager [60]. It manages and controls dual-band Wi-Fi (2.4/5 GHz), Dual-mode Bluetooth (4.1/5 GHz), and IEEE 802.15.4 (for Thread and Zigbee). Has ultra-low-power consumption (<50 μA), which is 25 times lower than competing solutions [61]. Two operation modes: Hosted Mode, also called n-LinkTM (has Wi-Fi stack, Bluetooth stack and profiles, and two interfaces), and Embedded Mode, also called WiSeConnectTM (has Wi-Fi stack, TCP/IP stack, IP modules, and Bluetooth stack built within the RS9116W chip). Does not have an interoperability feature between network stacks that allows packets to travel from one network source and arrive at a different network destination.
- SimpleLink™ Multiprotocol Wireless MCU: Multiprotocol devices equipped with Microcontroller Units (MCU) to foster concurrent multiprotocol operations and coexistence with other protocols [62]. (i) Concurrent Multiprotocol Single-chip: Allows a single radio to concurrently run multiple network protocols. Products are CC1352R, CC1352P, and CC2652R running Bluetooth LE 4.2 or Bluetooth 5.1, together with all IEEE 802.15.4 devices. (ii) Swapped Multiprotocol Provisioning: Allows devices to share or swap information such as device ID, security details, and network name (note: only possible with BLE). Products are CC1352R, CC1352P, CC2652R, CC3235SF, and CC3135 running BLE together with any network: Zigbee, Wi-Fi, or Thread; and (iii) Coexistence (Two-Chip Multiprotocol): Make use of time division multiplexing, and it allows two devices to share a single antenna without interference. Products are CC3235S and CC3235SF running BLE and Wi-Fi only.

Based on the hardware description provided in Section 2.2.3 above, it could be seen that the SimpleLink™ Multiprotocol MCU presents a unique approach to interoperability to improve connectivity within the home and industrial environments. Our review reveals that its swapped multiprotocol provisioning has an edge over the other multiprotocol hardware, because it allows Bluetooth LE to exchange information or data with any single protocol at a time. For example, Bluetooth LE can request a connection with Zigbee and share limited data packets but cannot connect with other network protocols such as Wi-Fi or Thread, while its connection with Zigbee remains active. To address this limitation, we developed JosNet, which allows a protocol to connect and share packet data with any other available network. Existing research, e.g., Reference [42], proposed a gateway (in the form of a raspberry pi) to promote interoperability between Zigbee and Bluetooth LE. However, they did not disclose details of their implementation procedure, technical configuration, and concrete evidence of interoperability. On the other hand, Reference [43] proposed the use of smartphones as a universal IoT gateway between the internet and smart IoT devices. IoT devices transmit packets over the internet using Bluetooth, Zigbee, and Wi-Fi, while the smartphone retrieves the data and forwards it to the target destination. The limitation in their research is that data packets are routed over the internet, which increases the latency time and reduces throughput, therefore causing a security risk of the packet data. To address this issue, our research provisions Machine-to-Machine (M2M) communication and presents routing protocols to monitor connected devices, with large-scale implementation of multiple connected devices. MQTT has been employed as a brokerage [63], which is connected to other interfaces, where each interface is linked to a wireless network. The MQTT brokerage stores data distributes and relays data to users. Their research claims to have provided a partial solution to address the interoperability challenge as raw data are collected by the microprocessor and converted into JSON-SenML format, which is “understood” by a different network radio. The limitation of their research is that they did not provide concrete performance-related evidence of the integrated system. Additionally, the partial implementation seems to imply that the brokerage has not been fully tested on different network protocols and platforms. Once again, our research assumes full implementation of the interoperability solution supported by evidence-based performances.

## 3. Methodology

**Figure 1:**
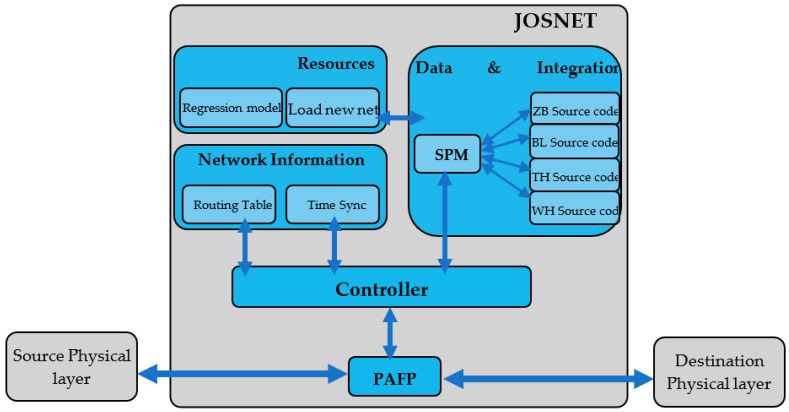
JosNet Stack Architecture.

This research adopts the “Design and Creation” strategy, which focuses on developing new information technology products known as artefacts [64,65]. Artefacts in computing could assume the form of a new piece of software application or data models, with the aim of solving the existing problem [66,67,68]. We present JosNet as an innovative piece of software construct designed to solve the existing problem. In this section, we shall discuss the following that are relevant for the development of a JosNet proof of concept: workflow process, software/hardware platform/framework, JosNet Stack Architecture, Network Sequence Diagram, and Development of JosNet. JosNet is developed using Microsoft Visual Studio, and its stack architecture is depicted in Figure 1.

### 3.1. JosNet Stack Architecture

In this research, a stack architecture has been adopted to model a workflow relationship between each framework and component of the system [69,70,71]. The components depicted in Figure 1 are Packet Arrival and Forwarding Point (PAFP), Resources Module, Data and Integration Module, Network Information Module, and Controller.

Packet Arrival and Forwarding Point (PAFP) is the point where packets or messages are received from the source together with information for the target destination. Packets or commands are next picked up by the Controller for processing and then forwarded through the appropriate COM port to the destination node. The Controller Module is in charge of handling packets, scanning for signals or message triggers, and processing commands. It is considered JosNet’s brain. As shown in Figure 1, the controller is directly linked to all the components of the system, except for resources that remain partially independent. The controller can request for routing table from all connected devices on the network at regular intervals to determine what port packets would be forwarded through. Communication with the port manager is also essential. The Data and Integration Services Module comprises the Serial Port Manager (SPM) and source codes. The Serial Ports Manager (SPM) is responsible for placing packets or data (together with other information requests) onto the physical medium and then wirelessly transferred to the next device. SPM connects with the source code of all network protocols to retrieve the ideal source code designed for each network protocol. For Windows OS older than Windows 10, it requires a hardware USB-UART driver to function properly. On the other hand, Source Codes are preinstalled manufacturer-specific configurations for each hardware device and its respective network protocol. They are connected to the Controller through the SPM, therefore providing the appropriate solution for each network technology when needed or requested by the Controller. The Network Information Module is another component of JosNet, which is built for the management and control of all connected devices across the network. It comprises Time Synchronisation, which ensures all devices on the network adopt the same clock as the host device to the precise millisecond. The Routing Table collects detailed information of every device on the network. Both Time Synchronisation and Routing Table are both connected to the Controller which uses such information to display results and determine the integration process, respectively. JosNet Resources are independent features built into the JosNet brokerage application but not directly related to the integration process of any WSN. They are Prediction Regression Model and adding new network technology onto JosNet. The Predictive Regression Model is built based on actual collected data (in CSV format) in this research to predict packet data size based on a given time value, while the packet delivery time is based on a given data packet size. The facility for uploading new technology fosters expandability, scalability, and robustness in JosNet brokerage. As the IoT world is ever-growing with a relentless call for emerging requirements of low-rate and low-power smart devices across the globe, new wireless technologies are constantly evolving. It is also a feature for future development of JosNet when the opportunity provides itself.

**Figure 2:**
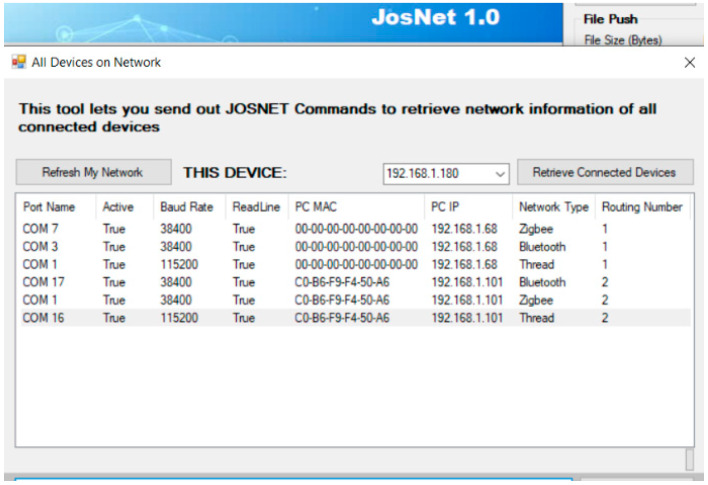
JosNet Routing Table.

Here, in this section, we shall provide details of JosNet’s Routing Table (see Figure 2). With the need for large scale JosNet implementation, it is imperative to keep track of all devices connected to the network for better management and message routing. Information such as station is linked to network protocol type, unique device ID or IP address, MAC address (if available), COM port number, routing number, and hops or stops from any point within the entire network. The Routing Table is, therefore, necessary for information collection, storage, retrieval, and updates. Additionally, it helps to implement message packet routing and hopping across multiple devices and stations only if integration is activated in JosNet brokerage.

The following steps explain the processes involved in retrieving devices information and routing number:

- Firstly, all JosNet brokerage stations require a local connection host (the same concept applied in Time Synchronisation) to keep information local without the need for internet connectivity and thus maintains the objectives of this research for machine-to-machine communication. We created a local Wi-Fi hotspot for all station PCs to connect;
- Next, the requesting PC sends a trigger containing its own information: “#JS|#GAD|#HOST=192.168.1.180:2020,#NET=ZB”. This is an array of four elements from the requesting station #JS, which represents “JosNet” as an initial declaration of identity. #GAD is the second element, which represents “Get All Devices” and retrieves all information from the SPM, which are COM port, network protocol affiliated to that port (displayed as network type), active status of the COM port, baud rate, readline status, and IP and MAC address of PC. The third element is #HOST, which represents the IP address and port number of the requesting/source station. The final element is “#NET”, which represents the requesting/source network type. The source network type from the above example is Zigbee. It is important to note that any trigger without the complete element would yield an error message;
- The trigger is then picked up by the Controller of other JosNet stations; it processes the request, retrieves the information (as stated in (b)) of all the connected devices via the SPM, and attaches its hop number before the response is sent only to the IP address of the requesting brokerage station;
- Information is then populated in the table, as shown in Figure 2 above.
- For the effective interoperability of connected devices, JosNet stations make use of the Routing Table for effective routing in the large-scale implementation of JosNet.

For small organisations or home automation with a limited number of devices, large-scale implementation is completely unnecessary, and therefore, routing across multiple JosNet brokerage stations is highly impracticable.

### 3.2. JosNet and Network Sequence Diagram

**Figure 3:**
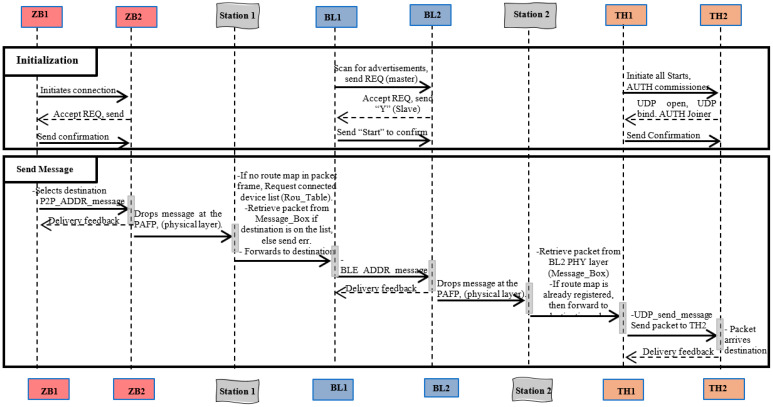
Sequence Diagram, Conversion, and Handing Over Process of the Connected Network Protocol on JosNet.

The JosNet Stack Architecture is related to the network sequence diagram depicted in Figure 3. Ref. Reference [71] defined a sequence diagram as a composition of event occurrences, lifelines, interaction fragments, messages, and combined fragments. Each element or component plays a pivotal role and helps to illustratively demonstrate every step in the system sequence. A sequence diagram is important in a WSN system, as it graphically showcases a dynamic representation of the system that could occur when objects interact to accomplish specified series of events or tasks [72]. It is the only model that shows the messages exchanged between the object class in the order in which the messages occur [72]. The network sequence diagram employed for Phases 1 and 2 of this research is depicted in Figure 3.

Phase 1 of Research:
  - At point ZB1: For initialisation, clicking “Activate Listen” on JosNet allows connected USB devices to initiate connection requests in search for any advertising device; this is the case for all network protocols connected to JosNet. However, in this case, ZB1 is an external device and not connected via USB to a JosNet station. After obtaining a response from ZB2, it sends all information, including the UART COM port, address ID, PANID, baud rate, signal channel, IEEE, and address. For sending a message, clicking the “Send” button on JosNet will load the packet message on the channel, with ZB2 as the route to its target address. “M2M_ADDR_message” indicates machine-to-machine communication, therefore designed for a specified device on the network.
  - At point ZB2: For Initialisation, regularly advertises for a connection request (REQ) and accepts REQ by sending “Y”, as well as sending its own device information (PANID, Device ID, channel, and more). For sending a message, packets are received from ZB1 together with information for the target destination, and it is responsible to drop off packets in the PAFP Module. Upon receiving the packet, ACK is sent back to ZB1 to confirm successful message delivery.
  - At Point Station 1: JosNet scans for indicators to determine how incoming or outgoing messages should be handled. For example, “P2P_ADDR_message” is an indicator that the incoming packet has one target destination and cannot be shared across the network (broadcast) with other devices until it has arrived at its destination and “O2P_ADDR_Message” indicates a broadcast message across the network. At Station 1, JosNet looks up the Routing Table of connected devices for the following reasons: firstly, if the target destination device is connected at any point in the entire network; secondly, if the target device understands the network protocol or it is preconfigured; and thirdly, to find the quickest routing hops or path to the destination. Packets cannot be forwarded if the destination is not found on the Routing Table, and integration is activated if the source network protocol is different from the destination.
  - Packets arrive at JosNet Station 1 via the physical layer of ZB2, and next, it reads all information or instruction included in the packet frame before forwarding them through the best-suited COM port to the appropriate destination (in this case, BL1 COM port). JosNet then triggers the sending command unique to Bluetooth device (BLE_ADDR_message); for Thread network, it is “UDP Send message”, and for Zigbee, it is “M2M” or “O2M”. These are unique triggers to the different network protocols. It is the responsibility of JosNet to automatically map out the correct routing path, at this point.
  - At point BL1: For initialisation, all Bluetooth nodes connected to the JosNet station play both master and slave roles to allow external Bluetooth devices to connect to the network. To establish a connection, BL1 scans for available beacons and, once found, assumes the role of a master and exchanges information such as Unique Identifier, physical location, and other required details. The message “Start” is then sent across to BL2 node to confirm the connection. For sending a message, JosNet forwards received packets through BL1 COM port using “BLE_ADDR_Message” to initiate this. BL1 then sends received packets to BL2, which is connected to JosNet Station 2. Note that the connection between Bluetooth node 1 (BL1) and Bluetooth Node 2 (BL2) maintains all connection protocols standard affiliated to the technology.
Phase 2 of Research:
  - At point BL2: For initialisation, BL2 is responsible for advertising its beacon from time to time and can also scan for other available beacons. BL2 becomes the slave when BL1 requests for a connection then sends a “Y” to accept the connection request (REQ). For sending a message, BL2 receives a packet message from BL1 and drops it off at the PHY layer accessible to JosNet Station 2. Upon receiving the packet, ACK is sent back to BL1 to confirm successful message delivery.
  - At point Station 2: Packet is retrieved from the PHY layer of BL2, and a check is carried out at this point by JosNet for the routing map and destination protocol. The routing map would help Station 2 place the packet on the right port for retransmission or forwarding, while knowing the destination protocol helps JosNet to carry out the right integration. Station 2 plays the same role as Station 1 (explained above). If a routing map is found, packets are then forwarded in the allocated direction; else, Station 2 sends a request for the list of devices on the network before forwarding the packet to the right destination.
  - At point TH1: For initialisation, a Thread commissioner device needs to be authenticated by sending a series of initialisation codes beginning with its network name, extPANID, and PANID. When a Joiner device is found, TH1 sends the master key, starts Thread, starts ifConfig, starts commissioner, and EUI64. A connection is established after TH2 responds with further initialisation. For sending a message, the Thread commissioner device (TH1) uses “UDP send message” to send packets to TH2 when initiated from JosNet. Messages can either start from this point or be delivered here for further forwarding to the designated destination.
  - At point TH2: For initialisation, the Joiner device also goes through an authentication process by starting up ifConfig and starting up Thread, Joiner ID, and the UDP bind port number, which are all sent to the commissioner device. After these are confirmed or verified by TH1, UDP is then started. NOTE that all initialisation codes with Thread network have been manually configured onto JosNet, as the OpenThread source code does not automatically provide this, giving room for programmers to configure this feature based on individual platform usage. Therefore, Thread network initialisation is configured, along with other integration processes. For sending a message, TH2 is the target destination (in this case) and therefore will not forward the message. Upon receiving the packet, ACK is sent back to TH1 to confirm successful message delivery.

It should be noted that communication can be initiated from any network protocol within JosNet brokerage, including Bluetooth, Thread, or Zigbee network. The above sequence is an example of communications initiated from Zigbee node 1 and travelling across JosNet Station 1 and Station 2 before arriving at Thread network node 2. Delivery feedbacks or ACK are not sent through JosNet but, rather, to the source node of the same network, as shown in the sequence diagram with reverse dotted arrow lines.

### 3.3. Test Deployment of JosNet

**Figure 4:**
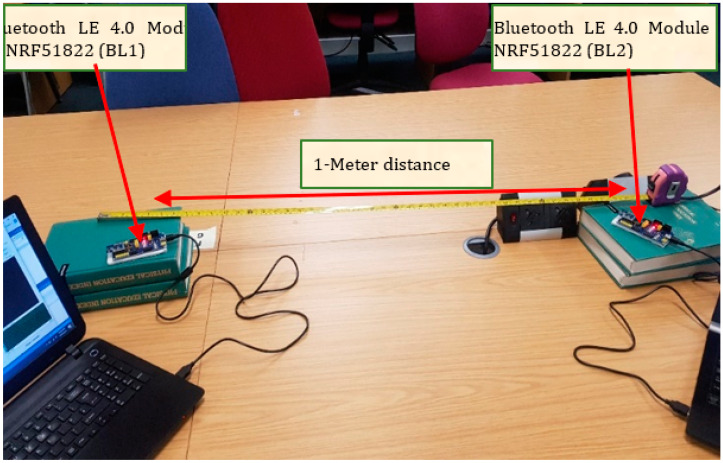
Respective Connection of Module Devices (BL1 source and BL2 destination) to PC1 and PC2.

**Figure 5:**
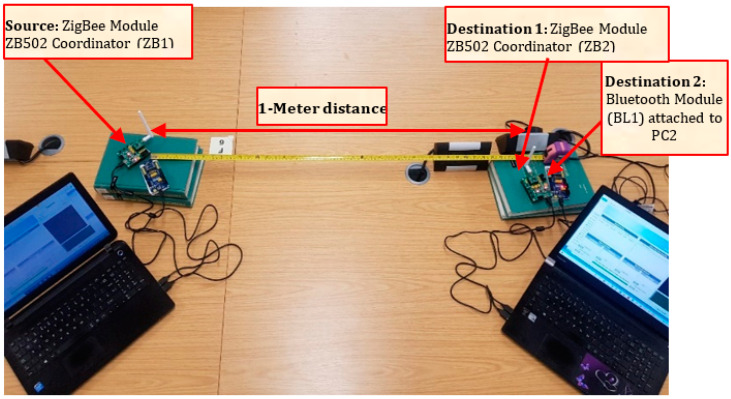
Respective Connection of Module Devices (ZB1 source and BL2 and ZB2 destinations) to PC1 and PC2.

Discussion of the test integration of Bluetooth, Zigbee, Thread, and WirelessHART is in this section. The hardware involved are Bluetooth Core51822 module and BLE400 Development Board (for Bluetooth network) and CC2530 Zigbee module and ZB502 development/Evaluation board (for Zigbee network). Several experiments have been carried out to evaluate the performance of existing ZigBee and Bluetooth technologies, as well as our proposed novel integration of BLE and ZigBee wireless networks. The experiment set-up comprises two separate computers (PC1 and PC2), as shown in Figure 4 and Figure 5 below. One ZigBee node (Z1) plays the role of a transmitting (source) device, while the second Zigbee node is the receiver (or destination) device (Z2). The transmitter is set to transmit preset data sizes in (8, 16, 32, 64, 128, and 256) bytes at a 2.4-GHz frequency. The power is not regulated or monitored but, by default, uses its built-in default factory standard power supply (maximum of 500-mA power supply) via a USB cable for each development board.

To test the interoperability of both ZigBee and Bluetooth LE network technologies, ZB1 module (source) is connected to PC1, while both ZB2 and BL2 (destinations) are connected to PC2 with the same power source and frequency as previously mentioned. It will be imperative to test their performances at a further distance of 10 m and have a wall barrier. In this case, we position each node with two rooms apart from each other. To determine the average quality of any wired or wireless network, it is imperative to test the integrated network multiple times with varying distances, different packet sizes, and wall barriers.

**Figure 6:**
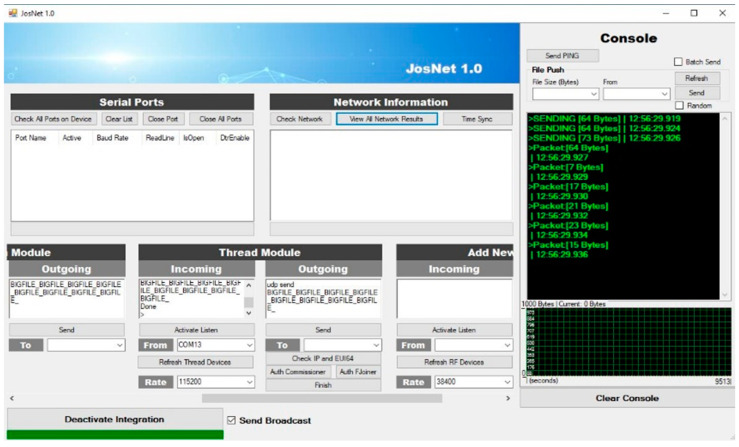
JosNet Interface (with graphs and console display) PC2 (destination), which receives data packets from PC1 (source).

JosNet brokerage application is used to initiate connections and make the integration between Zigbee, Bluetooth, Thread, WirelessHART, and other related network protocols possible, as we will discuss in the next section. To allow interoperability between ZigBee and Bluetooth LE, the “Activate Integration” must be turned on at the receiving (destination) computer (PC2), as shown below in Figure 6. It is necessary to activate Integration to allow the network interpreter to carry out the handing-over process and synchronise packets across every connected network node. For example, when Bluetooth LE sends a message from PC1 (source) to ZigBee on PC2 (destination), “Activate Integration” should be activated on PC2. Timestamp and data sizes are displayed on the console section of JosNet on the receiving PC (in our case, it is PC2).

As highlighted in the introduction of this paper, we will focus on devices that make use of low power and low rates to communicate or transmit a very small string of information in-line with the IEEE 802.15.4 industry standard [73,74]. The standard defines technical specifications, test conditions, receiver sensitivity, frequency, and transfer limits, which enact both national and international laws protecting the environment. Although BLE is not classed under IEEE 802.15.4 but, rather, under the IEEE 802.15.1 standard [25]; it is, however, necessary to include Bluetooth, as it is a major player in the IoT industry.

The network development boards used for JosNet implementation are shown below:

#### 3.3.1. CC2530 Zigbee Module and ZB502 Development/Evaluation Board

**Figure 7:**
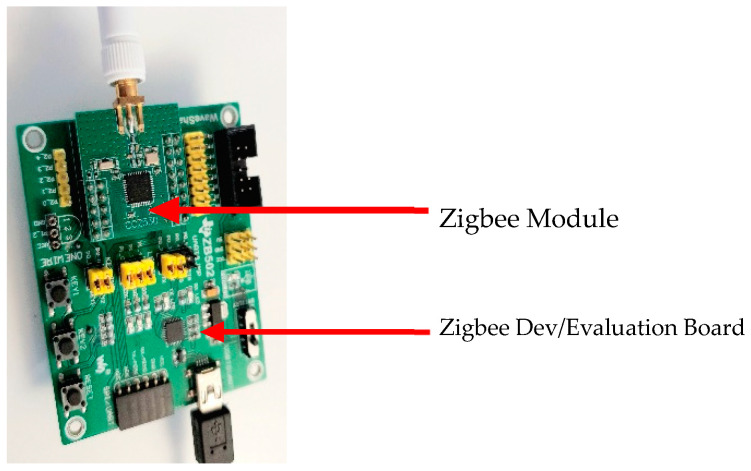
A CC2530 Zigbee module and ZB502 development board used to configure the Zigbee network during the research [75].

This device consists of a Zigbee module by Zigbee alliance attached to the development board by Waveshare (See Figure 7), and both together make one complete evaluation kit. Two evaluation kits are required for Zigbee connection, at least one coordinator and one router, while a third device can be added known as the end device. As expected, the CC2530 Zigbee module is designed to IEEE 802.15.4 standard specifications, which include a frequency range of 2.4 GHz, 250 kbps, AES 128 security coprocessing, collision avoidance feature, and CSMA/CA integration. The board designed by Waveshare give a better opportunity for users to explore the Zigbee protocols via various interfaces: USB interface, UART/SPI interface, debugging interface, and serial port RX/TX interface. Power can be supplied to the board either through a USB connection or battery, which can be found on the reverse side of the dev board and operates between 2 V and 3.6 V, to give a wider power supply voltage and operates at temperatures between −40 and 85 °C.

#### 3.3.2. Bluetooth Core51822 Module and BLE400 Development Board

**Figure 8:**
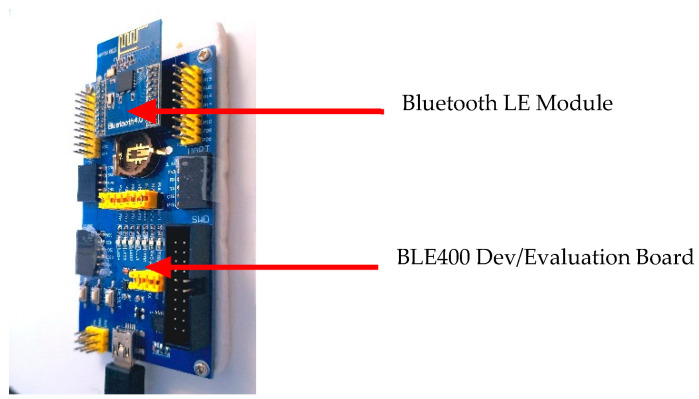
A Bluetooth core51822 module and BLE400 development board used to configure the Bluetooth LE network during the research [76].

Core51822 represents a wireless module system-on-chip (SoC) assembled by Waveshare in-line with the nRF51822 (see Figure 8) Bluetooth^®^ 4.0 multiprotocol wireless application, operating at 2.4-GHz radio frequency and suitable for low-energy and low-rate connections. It is referred to as a multiprotocol due to the wide range of deployment environments of the SoC, not because it can accommodate other wireless networks. A good advantage of the BLE400 board is that it provides an onboard battery holder, which allows for testing connections without cables and multiple interfaces like USB, UART, I2C, and SPI. Both the Zigbee CC2530 module and Bluetooth LE core51822 module are built with similar features that make them the perfect devices to build and test an interoperable network. These similar features include their operating frequency—2.4 GHz, baud rate—38,400, operating temperature—40~85 °C, programmable output power—+4 dBm to −20 dBm, and many more features on these boards. The motivation in selecting this product as the perfect fit for this research is that, as compared to other Bluetooth development boards, the BL400 is cost-effective with preconfigured SoC to work with.

#### 3.3.3. nRF52840-MDK IoT Development Kit

**Figure 9:**
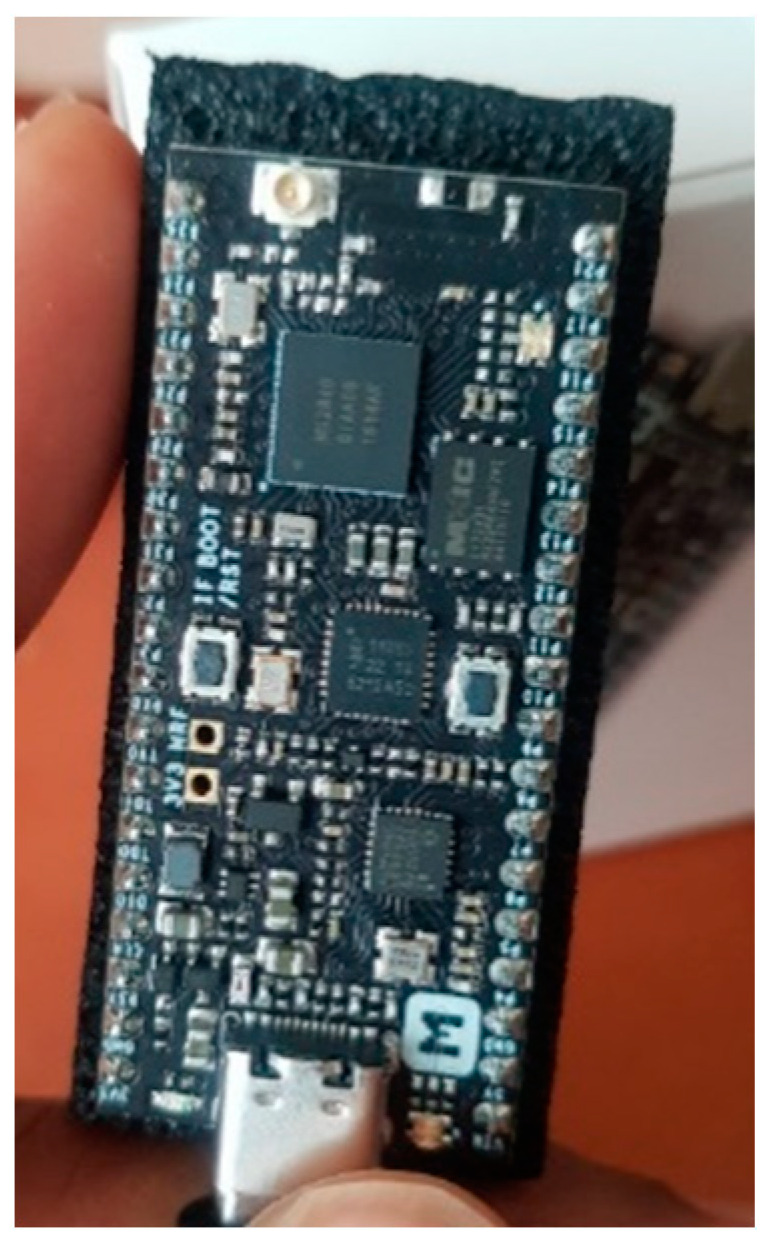
A nRF52840-MDK development kit used to configure the Thread network during the research [77].

A product from Makerdiary (see Figure 9), the low-cost nRF52840-MDK (Makerdiary Development Kit) is a versatile IoT hardware for low-energy and low-rate 2.4-GHz proprietary wireless network protocols using the nRF52840 system-on-chip [76]. The device is called versatile due to the multiprocessing capabilities of its Nordic SoC across various wireless technologies, including Bluetooth, Zigbee, Thread, ANT, WirelessHART, and other IEEE 802.15.4 network protocols with a 2.4-GHz frequency.

#### 3.3.4. Nordic nRF52840 Dongle

**Figure 10:**
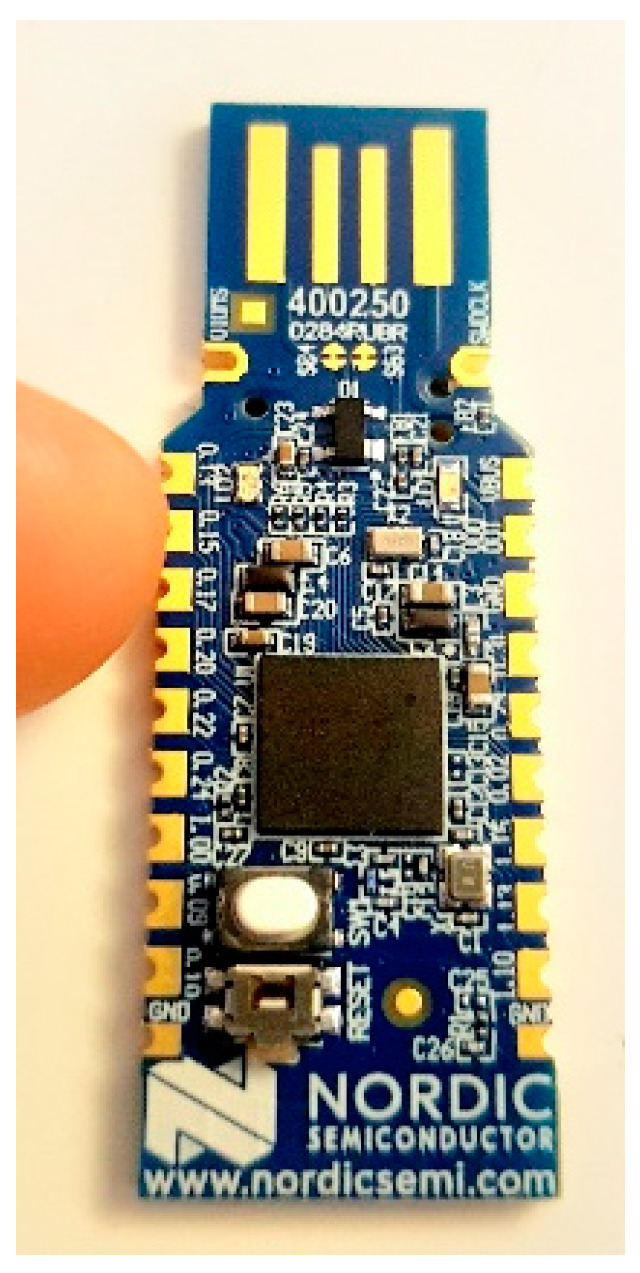
nRF52840 dongle used to configure the WirelessHART network during the research [78].

The nRF52840 dongle (see Figure 10) is a product from Nordic Semiconductor and built with the same SoC as the Makerdiary Development Kit and so plays almost the same role. This low-cost dongle device is compatible with Bluetooth 5, Bluetooth LE, Bluetooth Mesh, ANT, Zigbee, Thread, WirelessHART, and other 2.4-GHz wireless proprietary protocols (whichever is installed on it). The “nRF Connect for Desktop” is a PC application for adding a configuration onto Nordic devices, of which nRF52840 fits perfectly well [78].

### 3.4. Large-Scale Test Implementation of JosNet

**Figure 11:**
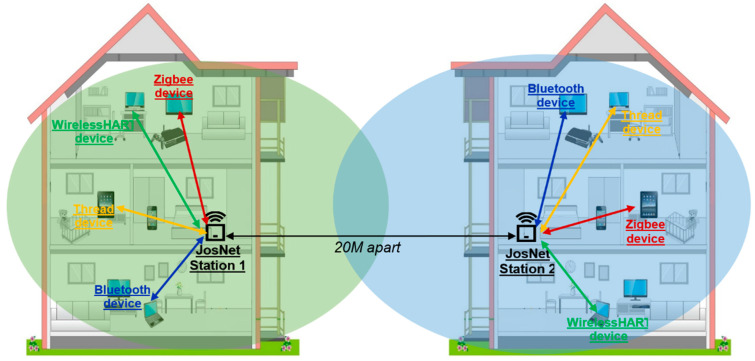
Structural representation of multiconnected buildings using JosNet.

**Figure 12:**
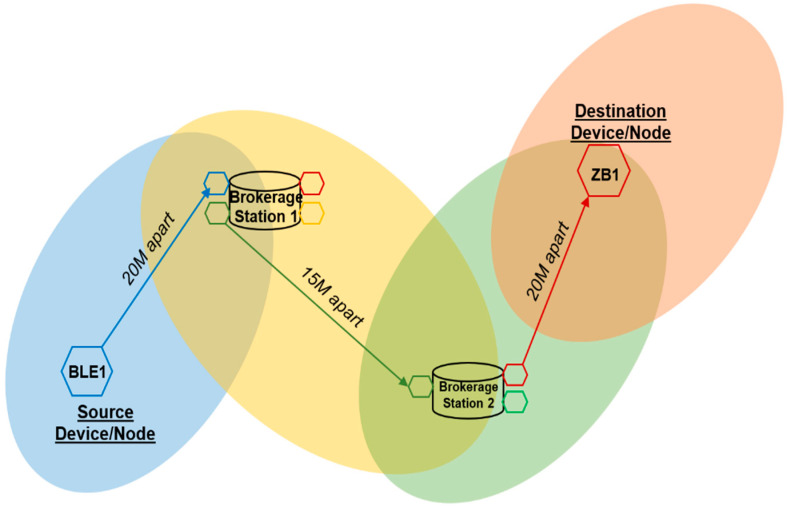
Communication between the BL1 node and ZB1 node.

Mesh network connectivity is extensively adopted in Zigbee, Thread, and WirelessHART and is strongly influenced by the employment of the IEEE802.15.4 wireless standard for connecting multiples devices accompanied with 6LoWPAN, which provides internet access to smart devices. The mechanism for header compression and encapsulation involved in 6LoWPAN allows packets to be sent or received using IPv4 and IPv6 transfer protocols. Before discussing the large-scale test implementation, we shall describe how a physical JosNet station (see Figure 11 and Figure 12) is set up and tested using the previously discussed hardware development boards. This part of the research validates and verifies how multiple network protocols can be integrated within a broker (i.e., JosNet) using a simplified interoperable network standard.

It is imperative that the JosNet brokerage is tested within a large-scale setting between two or more buildings (e.g., in a shopping mall or university campus environment). As a comparison, Wi-Fi boosters or extenders, sometimes called Wi-Fi repeaters, are a popular plug-in device used (in large buildings, office complexes, or commercial centres) to extend Wi-Fi radio coverage. It receives a Wi-Fi signal from the main router and then repeats the radio coverage. This provides a wider radius, thereby improving the network quality. Although JosNet brokerage does not repeat signals, as it sees every device on the network as an individual node, it applies the mesh routing principle when sending data packets across multiple points from source to destination. The similarity drawn from a repeater is that it extends the network coverage over a given area just like any mesh network. This would ensure that locations such as commercial buildings, multi-storey car parks, shopping malls, and large acres of farms can communicate seamlessly regardless of the network protocol deployed. The source device or sender must connect to the network through the IP address or device ID of the destination device or receiver to ensure the integrity of the service is not compromised.

**Figure 13:**
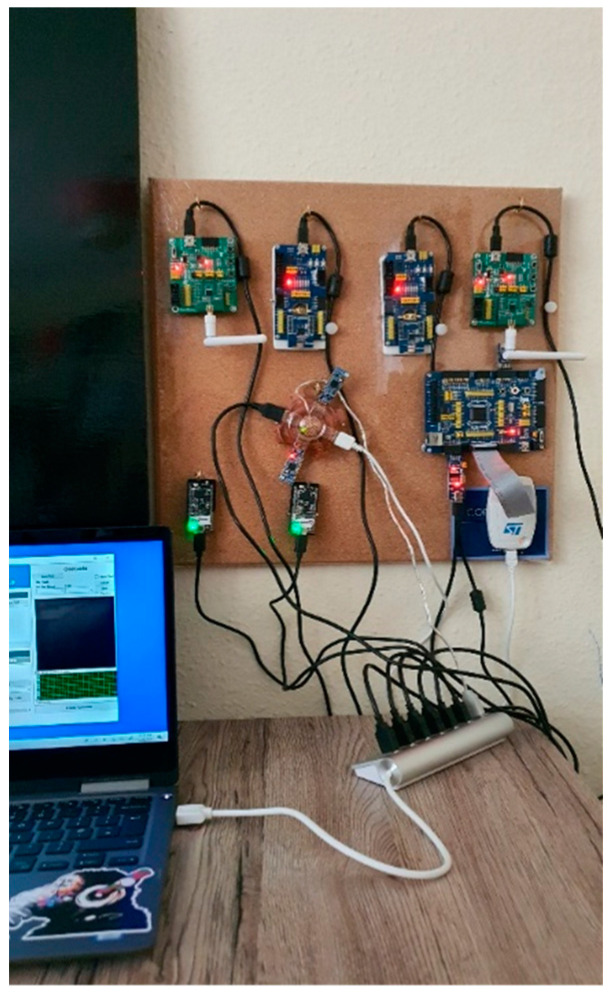
A JosNet Station Capable of Accommodating Multiple Network Technologies within a Single Station.

As shown in Figure 11 and Figure 12, a device using BLE (BL1) aims to communicate with a Zigbee device (ZB2) 55 m away either across buildings or within a large commercial building. We can assume that both buildings have JosNet brokerage installed as shown in Figure 13, and therefore, both devices can be connected through the nearest brokerage station. BL1 selects the destination ID or IP address, writes or attaches a message, then forwards it. An error message is displayed if ZB2 is not reachable; else, the Brokerage Station 1 (20 m away) receives the message via a Bluetooth port, and so, with the interoperability activated at Station 1, the message is translated and handed over to Thread, which subsequently forwards it to another Thread at Brokerage Station 2 (15 m away). Note that Brokerage Station 1 can forward packets through a different network protocol (e.g., Zigbee), which has similar routing points or a routing table (as shown in Figure 11 and Figure 12 below). Additionally, note that Brokerage Station 2 does not have a Bluetooth node, as packets are now handled through other nodes connected to JosNet. Next, Thread hands over the message packet to Zigbee at Brokerage Station 2, and finally, the receiving device or destination device (ZB1) receives the message from BL1 in less than 150 ms. The same concept is applied in a large network area (e.g., university campus, large forest area, shopping malls, or multistory car parks), where packets are transmitted across the network with the support of JosNet, only if the destination node is registered on the routing table. It is important that all communication protocols across the network are well-connected and updated in the routing table for faster and smooth routing. However, in situations where a specific network protocol is not available (e.g., the case here at Station 2), with JosNet interoperability capability, packets are transmitted as a broadcast message to all networks, thereby solving the problem by integrating both network packets.

## 4. Results and Evaluation

In this section, we present and discuss the range of experimental setups and results from the integration of wireless network protocols across different connection set-ups to evaluate the performance of our brokerage.

The following key will be used throughout this section:

End-to-end latency = time interval between departure time of first bit from source when the first bit leaves source to arrival time destination;
Date Loss = difference between sent file size and received file size;
Data Size = size of the data packet sent across the network from source to destination node;
Average Time = (T_1_ + T_2_ + … + T_n_)/n, where T represents time.

### 4.1. Experimental Setup

**Figure 14:**
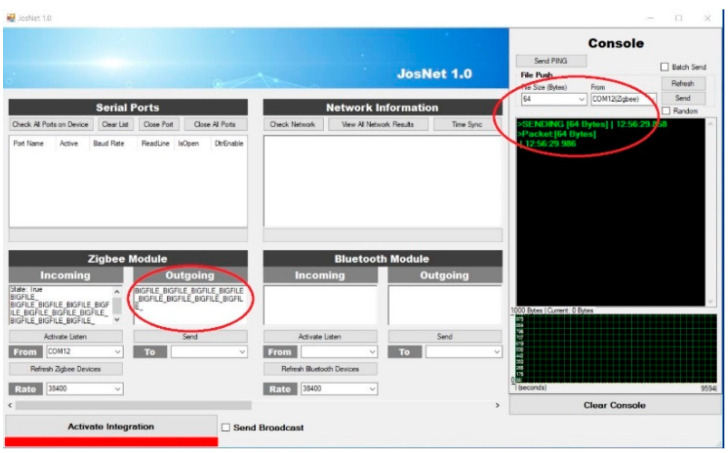
Source Zigbee external node (ZBx) sending 64 bytes of data through JosNet station 1 and station 2 to TH and BL.

**Figure 15:**
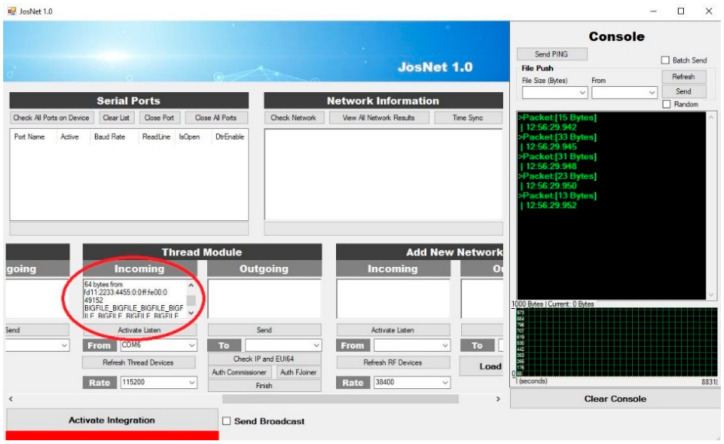
Thread device receives 64 bytes packet from Station 2.

**Figure 16:**
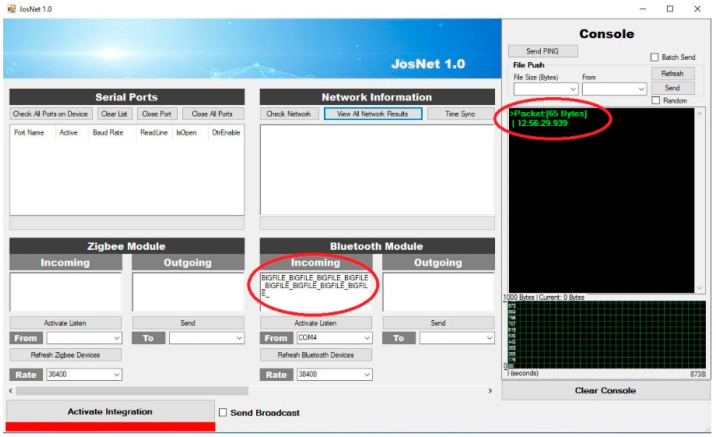
Bluetooth node (BL2) receiving 64 bytes of data from Station 2.

**Figure 17:**
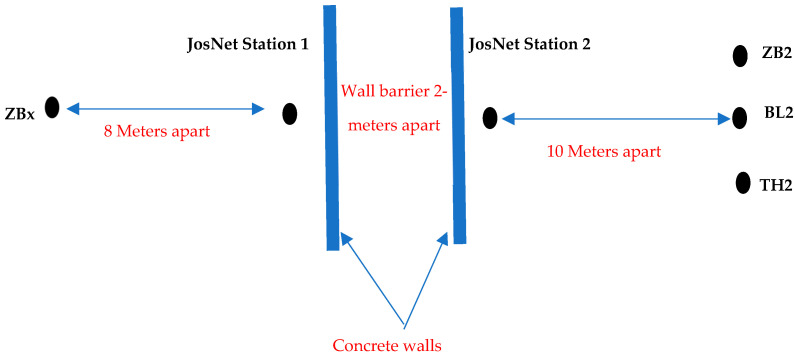
Schematic representation of the nodes separated by 2 concrete walls for the Zigbee–All connection pair.

Achieving the best results involved running the experiments multiple times (in this case, over 10 times) for every separate setup and connection pair, at different times of the day, and in varied environmental conditions. This varied environment is to replicate real-life conditions and ensure the proposed network nodes operates to standard. The type of varied environmental conditions being referred to involves a space with people walking around the equipment room and a reasonable noise level, with furniture like tables, chairs, and bookshelves. There are seven different experimental setups: Zigbee to Zigbee (ZB–ZB), Bluetooth LE to Bluetooth LE (BL–BL), Thread to Thread (TH–TH), Zigbee to Thread (ZB–TH), Thread to Zigbee (TH–ZB), Zigbee to Bluetooth LE (ZB–BL), and Zigbee broadcast to all connected network protocols (ZB–All). In Experimental Setup 7, it is a Zigbee Mesh Broadcast Message with a 20-m barrier wall (see Experiment Setup 7 below). This test is set up to embody the entire process at the same time, such that one network technology (in this case, Zigbee) sends a message to all the nodes in the network at a longer distance than usual, mesh routing across two brokerage stations, and wall barriers. The barrier represents a real-life building environment rather than a simulation. Here, the “Send Broadcast” option is activated at the brokerage, which indicates the message is not assigned for a specific device or node and, therefore, not private, as compared to Experimental Setup 6, Thread to Zigbee, where the “send broadcast” option is not activated and, therefore, assigned to a specific node device on the network. Some representative JosNet interfaces for the experimental setups are shown in Figure 14, Figure 15 and Figure 16 below, while the average for each test is then collected, analysed, and tabulated in Table 3. Figure 14 shows ZBx sending 64 bytes of data to all connected nodes, as through Station 2 to TH and BL (Figure 15) and BL2 receiving the packets (Figure 16). Figure 17 shows experimental Setup 7 for Zigbee broadcast to all available networks (ZB-All) through a wall barrier.

Experimental Setup 7: Zigbee Mesh Broadcast Message—20-m Wall Barrier.

### 4.2. Experimental Results and Discussion

Experimental results and discussion will focus on end-to-end latency and throughput. Some of the average transmission times are below 0.001 ms and, thus, could not be captured by JosNet. There must be sufficient data that allow a system to draw conclusions and apply relevant mathematical formulae or algorithms and make use of certain input features for prediction. Accurate prediction models are data-driven [36], and Wynants and colleagues [79] recommend conclusively that, to have an accurate predictive model, there must be at least 10 events per variable (EPV) and up to 50 events per variable (EPV) when variable selection is an option in the system. This research has ensured that at least 10 observations in every event or test set-up is carried out to ensure accuracy and consistency of the result outcomes generated. The averages of the repeated experimental runs (*n* = 10) are presented in Section 4.2.2.

#### 4.2.1. Average End-to-End Latency

In telecommunication, the transmission time is the amount of time from the beginning to end of the message transmission, while, in data communication, transmission time can be referred to as the time between the first bit leaving the sender and the last bit arriving at the destination [80]. In the JosNet brokerage application, the transmission time is captured by clocking the exact time each event occurs on the physical layer of both source and destination addresses.

End-to-End latency time is defined differently in the network communication spectrum. Reference [81] defined end-to-end latency as the amount of time required to transfer a bit of data from one point to another. Reference [82] also defined latency as the time incurred during transmission of a message from one designated node to another. Both definitions provide an acceptable premise for the results obtained in this paper, as JosNet captures the end-to-end latency time from the first initiated action to send a data packet until the last bit arrives at the destination node. End-to-end latency time is recorded to provide a performance metric and assess the validity of JosNet as a software communication medium for heterogeneous LrWPAN protocols.

Setting up devices with the same network protocol is to establish a benchmark for comparative purposes. For example, if the average latency time for BLE to BLE with a 100-bytes data packet is 0.04 ms, we can compare the outcome of Zigbee to Thread (JosNet brokerage integration) using the same parameters. For this test, the Bluetooth LE devices were placed 10 m apart, as shown in Figure 11 and Figure 12, each attached to a JosNet station (PC).

#### 4.2.2. Univariate Linear Regression Model

**Figure 18:**
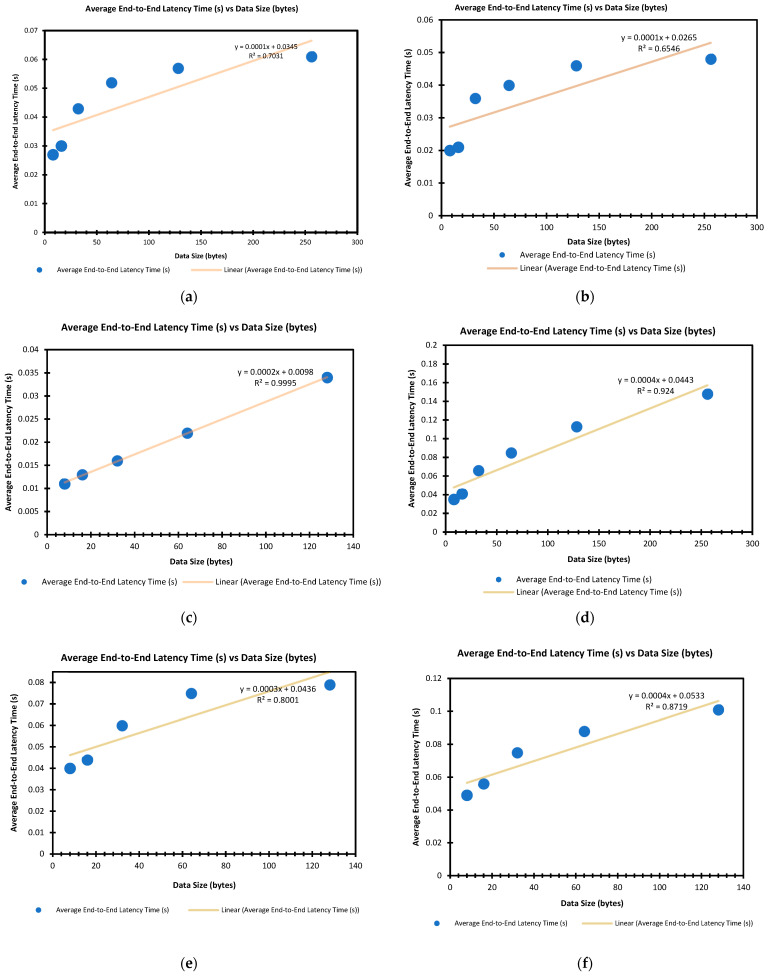

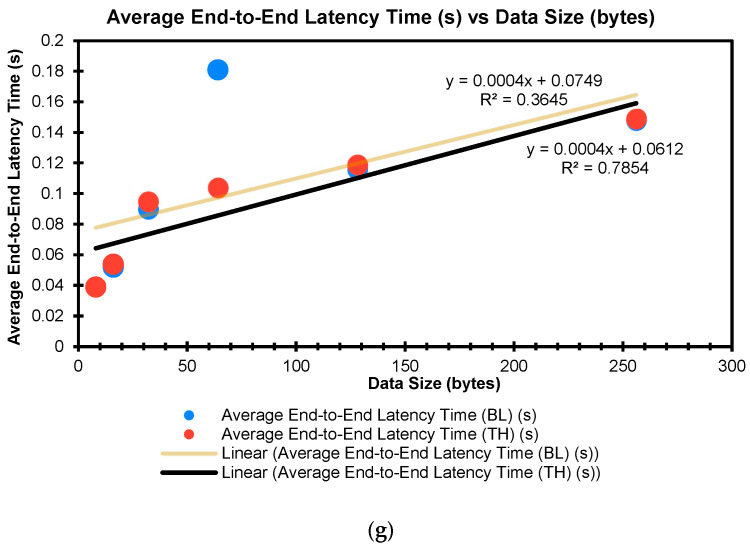
The graph for Average End-To-End Latency time against Data packet size for all connection pairs: (**a**) Zigbee to Zigbee; (**b**) Bluetooth to Bluetooth; (**c**) Thread to Thread; (**d**) Zigbee to Bluetooth; (**e**) Zigbee to Thread; (**f**) Thread to Zigbee; (**g**) Zigbee to all—Mesh broadcast (20 m range with wall barrier).

**Figure 19:**
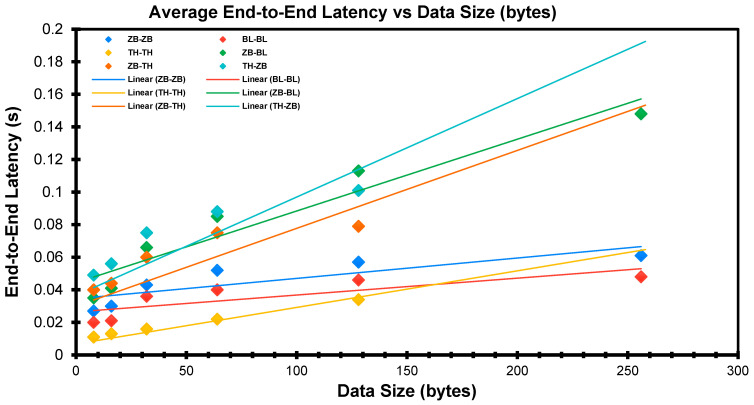
Combined End-to-End Latency Graphs for All Connection Pairs and Their Respective Regression Lines.

System modelling and evaluation provides an in-depth understanding of its performance [83]. This section will discuss the building of linear regression models for end-to-end latency versus data packet sizes. Figure 18 shows the graphs of average end-to-end latency time against data size, a combination of all graphs and their respective regression lines derived is then shown in Figure 19. Note that, in the below tables (Table 3), some results could not be captured, because they fell below 0.001 ms.

Regression analysis is the process of predicting values of a dependent variable based on the relationship or influence other independent variables have on the dependent variable. Reference [84] observed that a regression analysis mostly involves a complex diverse response (dependent variable) versus predictors’ (independent variable) relationship. To develop a robust and accurate prediction algorithm for the packet data size or prediction against time, the relationship between both variables must be well-understood, and the primary use of a regression analysis is to predict or forecast values from the existing data and find the correlation between them. Reference [85] defined a regression model as the relationship between a response or unknown variable (y) and other predictors x_1_, x_2_, …, x_n_, mathematically denoted as y = β_0_ + β_1_x_1_ + … + β_n_x_n_ + є, where β_0_, β_1_, …, β_n_ are the known variables, while є is a bias.

Based on the experimental outcomes, two salient variables, end-to-end latency (time) and packet data size, are captured, analysed, and employed to build latency-packet size regression models for the various communication protocols pairs (see Figure 19). The graph shows a plot of the average end-to-end latency time against the packet data size with the best-fitted linear trendline. The models are tabulated in Table 4 below.

Based on the results in Table 4, it could be seen the R-squared values for all the regression models were above 0.60, while two exceeded 0.90. For similar packet data sizes, the transmission latency for TH–ZB seemed to be the highest, while BL–BL seemed to be the lowest.

#### 4.2.3. Throughput

To ensure connected network nodes perform at the minimum average standard for a low-rate and low-power network, it is vital to measure the throughput based on the accepted criteria, establish what the criteria are, and discuss the results or outcomes from the integration of Zigbee, Thread, and Bluetooth LE. According to Reference [80], throughput is the measure of how fast data can be sent through a network and is heavily dependent on the bandwidth of the link or medium of transmission.

The formula for throughput (kilobits per second, kbps) = Data Size (bytes)/Transmission time (second) [86,87]. Thread and Zigbee networks are based on the IEEE 802.15.4 standard, which allows a maximum bandwidth of 250 kbits/s for devices transmitting with a 2.4-GHz frequency. However, this is not the case in reality due to multiple factors. A major factor is the transmission of the protocol overhead [88,89], while other factors could include the cable size, amount of energy supply (i.e., energy supply increase or decrease throughput), development board processing speed, PC monitoring capacity, environmental factors (e.g., temperature and humidity), and other wireless network interferences with a similar 2.4-GHz frequency [90]. Table 5 shows the computed average throughput based on packet size and transmission time.

Using the transmission time discussed in Section 4.2.1, we can derive the average throughput with the corresponding data size to ascertain the performance of the integrated network protocol as compared to the existing direct communication.

**Figure 20:**
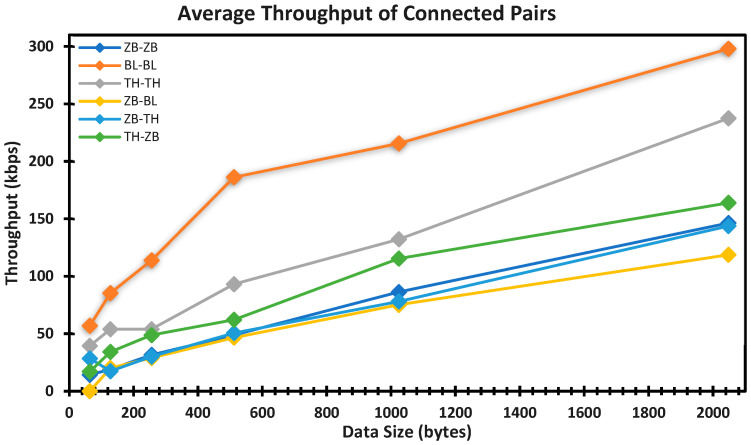
The graph shows a plot of the average throughput against data size with the best-fitted linear trendline. The regression models are tabulated in Table 6 below.

Note that the data size (bytes) is converted to bits per second (multiplied by 8) and then divided by the transmission time, which results in the corresponding throughput in bits per second; this is then converted to kbps (divided by 1000), as shown in Table 5. Average throughput is plotted against data size in Figure 20 below.

Based on the values in Table 6, we can show that the R-squared values for all the regression models fell between 0.90 and 0.99; this implies between 90% and 99% variation in the average throughput (Y), which is explained by the packet data size and transmission time of each connection pair. For the validation of JosNet brokerage, we shall compare the throughput performance derived from the regression model (depicted in Table 3 and Table 5) against the limited existing published results. Currently, there is a limited performance benchmark for JosNet. According to Silicon lab [91], this revealed that communication for M2M (or between a single hop) in a Thread network (assumed to be TH–TH) requires about 15 ms to send 50 bytes of the data packet (calculated throughput is 26.67 kbps), while two hops of the same data packet size (50 bytes) require about 22 ms (calculated to be 18.18 kbps). Based on the average throughput regression model for TH–TH, the computed throughput value for a data packet size of 50 bytes is 40.63 kbps and requires less than 13 ms. As for a Bluetooth LE network (assumed to be BL–BL), Silicon Lab [92] noted that the time for sending 300 bytes is 50 ms (calculated throughput is 48 kbps), while our JosNet average BL–BL throughput regression model yielded a value of 116.67 kbps and required 19 ms. However, the maximum bandwidth capacity at the physical layer of Bluetooth LE version 4.0 is 1 Mbps [93,94]. Finally, for a Zigbee network (assume to be ZB–ZB), Silicon Lab [95] revealed that the time for sending 300 bytes is 50 ms (calculated throughput is 48 kbps), and the result from our ZB–ZB throughput regression model was 32.92 kbps and required 70 ms. Reference [89] observed that the throughput for Zigbee to Zigbee falls between 47 kbps and 95 kbps. To date, there are no published results on the throughput of ZB–TH, TH–ZB, and ZB–BL.

## 5. Conclusions and Future Work

At the beginning of this research, we identified some existing gaps within Low-rate and Low-Power Wireless Personal Area Networks (Lr-WPAN) that form the current bedrock of IoT or smart technologies. The incompatibility of wireless network protocols has been the primary problem that results in a high cost of acquisition or maintenance, complexity, lack of reliability, in some instances, and high use of energy. To address this problem, we embarked on a novel implementation of a machine-to-machine software-based brokerage application (called JosNet) that can integrate low-rate and low-power wireless network technologies. It allows a specified network protocol to exchange data packets or commands with each other. This research encompasses a detailed review of existing technologies, the design and creation of JosNet brokerage, testing and retesting codes, and data collection and analysis, followed by the validation of JosNet. The following is a list of the key features or concepts that underlie our complex research approaches:

- Sequence Diagram: We discussed further specific rules put in place for the integration of all connected network protocols, and they are as follows: initialisation and sending a message in device connectivity, JosNet indicators—used by the controller to determine the source and destination of a packet message, routing map—JosNet controller makes use of routing map, which is displayed on the routing table as the hop number to find the shortest path to destination devices, and JosNet Stations, which reside between any two network protocols in the sequence illustration and represent the core of JosNet;
- JosNet stack Architecture: The stack architecture shows the relationship and workflow between different components of the system;
- Repeated testing: To ensure the consistency and accuracy of the data collected, the results are repeated during every network set-up to obtain an average reading from a minimum of ten trials;
- Different network protocol set-up: Setting up communication between each network protocol (discussed in this research) to ensure all network protocols can communicate between each other;
- Large-scale implementation and routing table: It would be oversimplistic to end without implementing integration with multiple nodes. Large-scale implementation ensures that the network coverage is wide enough for large areas such as university campuses, shopping malls, large farmland, forests, and other similar wide area coverages. To accomplish this, a routing table is essential.

Improving the performance while reducing the cost and energy consumption are the current norms in the tech industry. Regardless of the environmental factors, these are the expected delivery standards for new innovations. During the course of this research, our findings concluded that setting up communication in a controlled environment with enhanced testing equipment can greatly improve the performance and response time. Security and encryption on JosNet, which is an important subject alongside cost and energy, are beyond the scope of this research. We must reiterate that each network protocol maintains its security standards during communication between nodes; data packets are decrypted at the physical layer before the “conversion, handing-over, and integration” stage. The security of data packets is beyond this research scope and, thus, has not been investigated and discussed in detail.

Here, we describe the potential future of JosNet brokerage as an integration and interoperable platform for wireless networks. They are:

- Additional wireless network protocols to JosNet: A large number of network protocols or technology exists in the field of network communication regardless of the scope and delimitations of this research (limited to low-rate and low-power wireless networks); the addition of more wireless network protocols could create a robust system. JosNet has the potential to include other network technologies that transmit data in any form, as the same principle and standard applied during this research can be rebuilt, modified, or redesigned to share information within the confines of legal permits and moral standards. Information sharing is limitless in light of today’s high-speed connectivity; therefore, we envisage other network standards such as Wi-Fi, Sigfox, z-wave, LoRa, WirelessHart, etc. can be added to JosNet brokerage in the future to enhance interoperability;
- JosNet as a Hardware Device: Currently, JosNet is a piece of software application. It can be incorporated into a hardware device that can serve the same purpose within homes, offices, industries, the local community, and other commercial settings. This could provide users with the option of deciding what is best-suited for their environment or building settings. However, due to the limited resources available for this research, this cannot be possible. We used USB-connected cables to capture the activities of data packets throughout this research; this may not be practically sensible for JosNet stations to operate with PC/laptops or any other device with connected USB cables, as demonstrated;
- Security and Encryption: As mentioned earlier in the critique above, there is currently no encryption algorithm in place within JosNet to monitor packets at the PAFP before being forwarded to the next routing hop or device, as the case may be. Information such as the packet message, device IP and ID, routing table, and information included in each data frame may be captured, although very unlikely, as JosNet remains a physical station. Future work on data encryption should ensure this addition;
- Message sending for multiple selected devices: Currently, JosNet can only send to either one device node or the entire network using a “broadcast” message, regardless of the network protocol. In the future, we hope to extend the capacity of JosNet to send private messages to two nodes or more devices without broadcasting across the entire network. For example, if we have 50 connected devices on the entire network, a feature that allows users to send packet messages to only five devices would be desirable, without sending them to the 45 other devices.

## Figures and Tables

**Table 1:** Low-Rate Network Standards and Protocols (relevant for this research).

	Zigbee	Thread	WirelessHART		Bluetooth LE
OSI Reference Model				BL Protocol Stack	
**Application Layer**	ZDO	User defined	HART7, HART AL, EDDL	**APPS Layer**	Higher Layers APP (Application profiles and Services)
**Session Layer**	AES 128-bit encryption	SHA-256	AES 128-bit encryption	**HOST Layer**	GAP (Generic Access Profile) GATT (Generic Attribute Profile)
**Transport Layer**	UDP	UDP + DTLS	UDP		
**Network Layer**	Ad-hoc On-Demand Distance Vector (AODV)	Distance Vector Routing (DVR), IPv6, 6LoWPAN	6LoWPAN, TDMA		ATT (Attribute Protocol), SMP (Security Manager)
**Data Link Layer** **(DLL)**	IEEE 802.15.4 MAC	CSMA/CA	IEEE 802.15.4 MAC		L2CAP (Logical Link Control and Adaptation Protocol)
				**CONTROLLER Layer**	Link Layer (LL)
**Physical Layer** **(PHY)**	IEEE 802.15.4 PHY	IEEE 802.15.4 PHY	IEEE 802.15.4, DSSS		BL PHY (Physical Layer)

**Table 2:** Parameters of Low-Rate Network Protocols.

Parameters	Zigbee	Thread	WirelessHART	BLE
RF Band	868/915/2400 MHz	2.4 GHz	868/915/2400 MHz	2.4–2.48 GHz
Transfer Rate	20/40/250 kbps	250 Kbps	20/40/250 kbps	125/500/1000/2000 kbps
Range	10–100 m	100 m	10–100 m	Up to 100 m
Channel Width	5 MHz	5 MHz	5 MHz	Forty 2 MHz channels
Error Control	Cyclic Redundancy Check (CRC)	Cyclic Redundancy Check (CRC)	16-bit CRC and ACK (Acknowledgement)	Cyclic Redundancy Check (CRC)
Maximum Nodes	65,536	511	50–100	32,767

**Table 3:** The values for Average Transmission Time and Average End-To-End Latency Time for each connection pair: (**a**) Zigbee to Zigbee; (**b**) Bluetooth to Bluetooth; (**c**) Thread to Thread; (**d**) Zigbee to Bluetooth; (**e**) Zigbee to Thread; (**f**) Thread to Zigbee; (**g**) Zigbee to all—Mesh broadcast (20 m range with wall barrier).

**Data Size (Bytes)**	**Average Transmission Time (s)**	**Average End-to-End Latency Time (s)**	**Data Size (Bytes)**	**Average Transmission Time (s)**	**Average End-to-End Latency Time (s)**
8	-	0.027	8	-	0.020
16	-	0.030	16	-	0.021
32	-	0.043	32	0.005	0.036
64	0.036	0.052	64	0.009	0.040
128	0.054	0.057	128	0.012	0.046
256	0.065	0.061	256	0.018	0.048
**(a)**			**(b)**		
**Data Size (Bytes)**	**Average Transmission Time (s)**	**Average End-to-End Latency Time (s)**	**Data Size (Bytes)**	**Average Transmission Time (s)**	**Average End-to-End Latency Time (s)**
8	0.005	0.027	8	-	0.035
16	0.007	0.030	16	-	0.041
32	0.007	0.043	32	-	0.066
64	0.013	0.052	64	-	0.085
128	0.019	0.057	128	0.051	0.113
256	-	-	256	-	0.148
**(c)**			**(d)**		
**Data Size (Bytes)**	**Average Transmission Time (s)**	**Average End-to-End Latency Time (s)**	**Data Size (Bytes)**	**Average Transmission Time (s)**	**Average End-to-End Latency Time (s)**
8	0.006	0.040	8	-	0.049
16	0.007	0.044	16	-	0.056
32	0.008	0.060	32	-	0.075
64	0.018	0.075	64	0.030	0.088
128	0.059	0.079	128	0.030	0.0101
256	-	-	256	-	-
**(e)**			**(f)**		
**Data Size (Bytes)**	**Average Transmission Time (s)**		**Average End-to-End Latency Time (s)**		
	**TH**	**BL**	**TH**	**BL**	
8	0.005	-	0.039	0.039	
16	0.006	-	0.054	0.052	
32	0.011	-	0.095	0.090	
64	0.069	-	0.104	0.181	
128	0.105	-	0.119	0.116	
256	0.163	-	0.149	0.148	
**(g)**					

**Table 4:** Univariate Regression Models Built Based on End-to-End Latency Experimental Outcomes.

Connection Pairs	Regression Model End-to-End Latency Model	R^2 ^Value
ZB–ZB	End-to-End Latency (Y) = 0.0001 × DS + 0.0345 (A)	0.7031
BL–BL	End-to-End Latency (Y) = 0.0001 × DS + 0.0265 (A)	0.6546
ZB–TH	End-to-End Latency (Y) = 0.0003 × DS + 0.0436 (A)	0.8001
TH–ZB	End-to-End Latency (Y) = 0.0004 × DS + 0.0533 (A)	0.8719
TH–TH	End-to-End Latency (Y) = 0.0002 × DS + 0.0098 (A)	0.9995
ZB–BL	End-to-End Latency (Y) = 0.0004 × DS + 0.0443 (A)	0.9240

Note: ZB—Zigbee; BL—Bluetooth; TH—Thread; ALL—all protocols in the experiment; DS—Data Packet Size; A—bias (this value is subject to the experimental conditions or external factors).

**Table 5:** Analysis of Average Throughput Performances with their Corresponding Connection Pairs.

Average Throughput (kbps)						
Data Size (Bytes)	ZB–ZB	BL–BL	TH–TH	ZB–BL	ZB–TH	TH–ZB
64	14.222	56.889	39.385	-	28.444	17.067
128	18.963	85.333	53.895	20.078	17.356	34.133
256	31.508	113.778	53.895	28.845	30.118	48.762
512	48.762	186.182	93.091	46.545	50.568	62.061
1024	86.232	215.579	132.129	75.156	78.019	115.38
2048	146.286	297.891	237.449	118.725	143.719	163.84

**Table 6:** Univariate Regression Models Built Based on the Average Throughput.

Connection Pairs	Regression Model Average Throughput (kbps)	R^2^ Value
ZB–ZB	Throughput (Y) = 0.0666 × DS + 12.939 (A)	0.9957
BL–BL	Throughput (Y) = 0.1137 × DS + 82.564 (A)	0.9058
TH–TH	Throughput (Y) = 0.0981 × DS + 35.722 (A)	0.9945
ZB–BL	Throughput (Y) = 0.0553 × DS + 11.039 (A)	0.9599
ZB–TH	Throughput (Y) = 0.0617 × DS + 16.558 (A)	0.9885
TH–ZB	Throughput (Y) = 0.0718 × DS + 25.290 (A)	0.9651

Note: ZB—Zigbee; BL—Bluetooth; TH—Thread; DS—Data Size (bytes); A—bias (this value is subject to the experimental conditions or external factors).

## Data Availability

The data presented in this study are available on request from the corresponding author.
